# The value of INnovative ICT guided disease management combined with Telemonitoring in OUtpatient clinics for Chronic Heart failure patients. Design and methodology of the IN TOUCH study: a multicenter randomised trial

**DOI:** 10.1186/1472-6963-11-167

**Published:** 2011-07-13

**Authors:** Arjen E de Vries, Richard M de Jong, Martje HL van der Wal, Tiny Jaarsma, Rene B van Dijk, Hans L Hillege

**Affiliations:** 1 Thoraxcenter, Department of Cardiology, University Medical Centre Groningen, Groningen, The Netherlands; 2 Martini hospital Groningen, Department of Cardiology, Groningen, The Netherlands; 3 Linköpings universitet, Department of Social- and Welfare Studies, Norrköping, Sweden

## Abstract

**Background:**

Although the value of telemonitoring in heart failure patients is increasingly studied, little is known about the value of the separate components of telehealth: ICT guided disease management and telemonitoring. The aim of this study is to investigate the value of telemonitoring added to ICT guided disease management (DM) on the quality and efficiency of care in patients with chronic heart failure (CHF) after a hospitalisation.

**Methods/Design:**

The study is divided in two arms; a control arm (DM) and an intervention arm (DM+TM) in 10 hospitals in the Netherlands. In total 220 patients will be included after worsening of CHF (DM: N = 90, DM+TM: N = 130). Total follow-up will be 9 months. Data will be collected at inclusion and then after 2 weeks, 4.5 and 9 months. The primary endpoint of this study is a composite score of: 1: death from any cause during the follow-up of the study, 2: first readmission for HF and 3: change in quality of life compared to baseline, assessed by the Minnesota Living with Heart failure Questionnaire. The study has started in December 2009 and results are expected in 2012.

**Conclusions:**

The IN TOUCH study is the first to investigate the effect of telemonitoring on top of ICT guided DM on the quality and efficiency of care in patients with worsening HF and will use a composite score as its primary endpoint.

**Trial registration:**

Netherlands Trial Register (NTR): NTR1898

## Background

Heart failure (HF) is the most common hospital discharge diagnosis in elderly patients [1]. Between the age of 70 and 80 years the incidence of HF is 10 to 20%. HF is associated with high mortality and morbidity, readmission rates and costs [1]. The readmission rates vary between 25% and 50% within 6 months after the first hospitalisation for HF, with a higher readmission rate within the first month after discharge[2,3]. The costs related to HF contribute to 1-2% of all healthcare expenditures and are mainly the result of hospital stay [4-6]. Because of an increasing shortage of resources, HF is a major public health problem and therefore, a more effective and efficient organisation of care for HF patients needs to be reconsidered. A first step in organising treatment and care for patients with chronic HF more efficiently, was the implementation of specialised outpatient HF clinics. In the recent European Society of Cardiology (ESC) guidelines, HF management programmes are strongly recommended for all patients with HF [1] and HF clinics are considered as 'usual care' in several European countries [7]. A widely used way to implement HF management is the use of specific disease management (DM) programs.

DM can be defined as an intervention, designed to manage a chronic disease and to reduce hospital readmissions, using a systematic approach to care and potentially employing multiple treatment modalities [8]. Control and cost effectiveness are substantial components of a DM program. Randomised studies suggest that DM programs can reduce readmissions for HF or cardiovascular disease with 30% [7,9,10] and significantly decrease mortality rates [11]. Yu et al [12] described that DM for HF patients, as recommended by the ESC guidelines, [1] are effective in reducing hospital readmissions and mortality rate [13]. However, inconsistent findings for readmission and mortality rates have been found, probably due to the variety of components and practical applications of the DM programs.

We recently reported results of the COACH study, a study on the effect of a nurse led DM program on clinical outcome [14], in which the positive effects of a DM program on readmission were not confirmed, although there was a trend to a reduction of mortality in the intervention groups. The INH study [15] on the effect of DM in HF, showed that a DM program compared to usual care could reduce mortality but not hospitalisation rates. Important components of this program were patient education, optimisation of medical therapy, psychosocial support and an easy access to healthcare. An important aspect for the treatment of HF patients is the prescription of HF related medication at an optimal dose i.e. ACE-inhibitors, beta-blockers, and aldosteronantagonists. The up titration to optimal dosage is an aspect that often takes place at a HF outpatient clinic. However, data from the Euro Heart Failure Survey showed us that guideline adherence for HF medication although improving still is not optimal[16]. In the IMPROVE study, dedicated HF clinics were associated with greater use of cardiac resynchronisation therapy and a better HF education, but not with better guideline adherence to medication [17]. Health information technology, integrated into a DM program might facilitate adherence to guidelines of health professionals [18]. With new information and communication technology (ICT), healthcare providers can be supported in the diagnosis, treatment and follow up of HF patients by expert computerised systems, based on guidelines and protocols [19]. These systems can be used to optimise medication according to guidelines and provide structural support and education [20]. We were the first to report promising findings on ICT guided DM in terms of higher doses of recommended HF medication and lower readmissions [21,22]. Another promising ICT tool is telemonitoring. Telemonitoring is often used to monitor patients at home and guide patients to take action in case of deterioration, but it also can be used to up-titrate medication according to guidelines at distance [23]. There is support that remote monitoring of patients with HF can reduce hospitalisation and mortality rates, [24,25] however results on clinical outcome and efficacy are inconclusive and limited [26-28]. There are also recent study's that where not successful in their primary endpoints [29,30]. Furthermore, cost-effectiveness of these systems has not been thoroughly evaluated. It can be concluded that the overall effects of telemonitoring are inconclusive. To summarise, due to a growing population of patients with HF and an expected shortage of healthcare providers in the near future, there is a need to seek to more cost effective and efficient ways of providing optimal care for HF patients, including a better adherence to guidelines. ICT guided DM tools in combination with telemonitoring could be of important value [31,32]. At the same time there is substantional data that the adaptation and implementation of those systems is lacking [33]. The experiences with such a system however are fragmented. User resistance is described as a major obstacle in the adoption of these computerised tools. More insight in user resistance and experienced barriers in using ICT guided DM tools is needed to successfully implementing such tools [34].

The IN TOUCH study will investigate the effect of telemonitoring in addition to an ICT guided DM system on the quality and efficiency of care for patients after worsening HF. This is the first study investigating a combination of two newly developed ICT interventions in a group of chronic HF patients on clinical outcome, adherence to guidelines, cost effectiveness and quality of life.

This study will add important information to other telemonitoring studies because of its strong commitment to ICT guided DM, the chosen composite endpoint, a strong focus on cost-effectiveness and the investigation of the influence of user aspects as resistance and barriers that accompany the use of modern healthcare related ICT tools.

## Methods/Design

### Study hypothesis

Telemonitoring added to ICT guided DM improves prognosis and quality of life in patients with HF compared to ICT guided DM alone.

### Aim of the study

The primary aim of this study is to assess the effect of telemonitoring on top of an ICT guided DM system in patients after worsening HF on the combined endpoint of death, readmission and quality of life, compared to patients treaded with ICT guided DM alone.

Secondary aims of the study are;

• To assess the effect of telemonitoring in addition to an ICT guided DM system compared to an ICT guided DM alone on the separate components of the combined endpoint (death, readmission and quality of life).

• To determine the cost benefit ratio of ICT guided DM with telemonitoring compared to ICT guided DM alone.

### Study design

**Figure 1:**
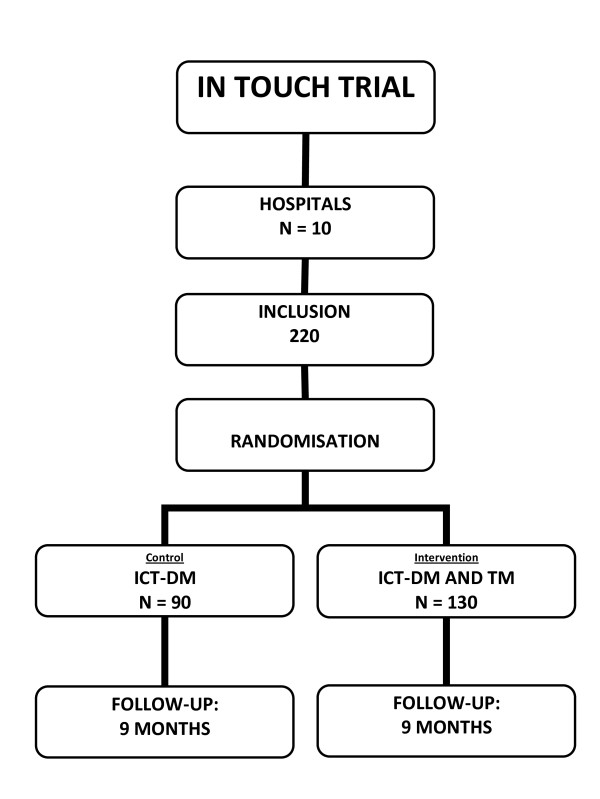
Flowchart of the inclusion.

A multicentre, randomised study in which in total 220 HF patients (NYHA II-IV) will be included. Patients will be randomised to the ICT guided DM (control) arm (N = 90) or into the ICT guided DM with telemonitoring (intervention) arm (N = 130) (Figure 1).

### Study population

All patients admitted to the intensive care/coronary care unit or cardiology ward for HF or visiting the outpatient clinic with worsening HF who need treatment or adjustment of oral or intravenous diuretics, can be included in the study. Other inclusion criteria are; evidence of structural underlying heart disease, left ventricular ejection fraction ≤ 45% and age of at least 18 years. Reasons for exclusion are myocardial infarction in the past month, cardiac invasive intervention (percutaneous coronary intervention, coronary arterial bypass, valve replacement, heart transplantation, or cardiac resynchronisation therapy) in the past 6 months or planned in the next 3 months, weight > 200 kg, actual haemodialysis and the use of other telemonitoring systems (Table 1).

**Table 1:** In and exclusion criteria IN TOUCH

***Inclusion criteria***
Worsening HF defined as signs of fluid retention (peripheral oedema/congestion) needing an increase of the dose of diuretics (i.v. or oral)
Evidence for structural underlying heart disease
Documented reduced left ventricular ejection fraction (LVEF) ≤45%
18 years of age
Patients have to be able to understand content of and willing to provide informed consent.
***Exclusion criteria***
History of myocardial infarction in the previous month
Life expectation less than 1 year
Undergone cardiac invasive intervention within the last 6 months (PCI, CABG, HTX, valve replacement, CRT implantation)
A planned procedure for PCI, CABG, HTX, CRT implantation or valve replacement in the following 3 months
Evaluation for heart transplantation prior to or during the study
Weight more than 200 kilogram
The use of telemonitoring devices at home
Haemodialysis

### Primary endpoint

The primary endpoint of the study is a composite, weighted score consisting of values for mortality, HF readmission and change in quality of life between end of the study and baseline measured with the Minnesota Living with HF Questionnaire (MLHFQ), adapted from the A-HeFT study [35] (Table 2). A readmission for HF is defined as an overnight hospital stay for HF or directly related to HF. The readmissions for HF will be blinded adjudicated by an endpoint committee. When data on quality of life are missing, the worst-case score for that component of the composite endpoint will be used in the analysis.

**Table 2:** score system for primary endpoint IN TOUCH

End point	Score
Death (at any time during study)	-3
Survival to end of study	0
First readmission for heart failure	-1
No readmission for heart failure	0
	
*Change in quality of life at 9 months*	
Improvement ≥ 20 units	+2
Improvement by 10 until 19 units	+1
No improvement by -9 until +4 units	0
Worsening by +5 until +9 units	-1
Worsening by ≥ 10 units	-2
	
*Possible score*	-6 to +2

### Secondary endpoints

Secondary endpoints of the study are the separate components of the primary endpoint. Other secondary endpoints are the total number and duration of all hospital admissions, treatment according to the guidelines using the criteria of the Guidelines Adherence Indicator-3 [36], number of visits to the outpatient HF clinic, patient and carer satisfaction, and cost-benefit ratio.

### Ethics statement

On March 2009 this study design has became ethical approval (M09.070323) given by the medical ethical commission (MEC) of the medical university of Groningen (UMCG).

The study has started (first patient) in December 2009.

### Incremental cost-effectiveness ratio (ICER)

Costs A distinction will be made between intervention costs and resource utilization costs. The intervention costs consist of the costs of the DM system and the costs of the telemonitoring devices and will be calculated as a lump-sum over the study's follow-up period. Resource utilisation costs will be estimated by preparing a structured data collection form to collect detailed information regarding scheduled and non-scheduled outpatient clinic visits and hospital admissions (both HF and non-HF related), ward type (e.g. intensive/coronary care unit, cardiology, general internal medicine), and cardiovascular procedures/operations. In addition, a patient questionnaire will be administered at 4.5 months and 9 months of follow-up to collect complementary data on general practitioner visits, home care utilization, and nursing home admissions. Unit costs will be estimated by using the Dutch guidelines for cost calculations and inflated to current price levels using a general consumer price index. Indirect costs, such as productivity losses, will not be taken into account.

#### Quality Adjusted Life Years (QALYs)

Preference-based quality of life scores will be obtained by administering the EQ-5D to all patients in both the control group and the two intervention groups at baseline, 4.5 months of follow-up, and 9 months of follow-up. QALYs will subsequently be estimated by calculating the area of the two trapezoids that result from linear extrapolation of the three quality of life scores.

#### Cost-effectiveness

The balance between costs and effects will be assessed by estimating the incremental cost per QALY gained (ICER) for the intervention group compared with the control group.

The time horizon over which the costs end effects of the different treatment strategies will be compared is equivalent to that observed during the period of the study (i.e. no future projections will be made). Uncertainty surrounding the ICER will be represented through the use of cost-effectiveness acceptability curves, which show, for each possible value of λ (i.e. the societal willingness-to-pay for one additional QALY), the probability that the intervention will be cost-effective.

### Randomisation and data collection

Patients can be randomised during admission for HF, and in case of worsening of HF at the outpatient clinic. After confirmation of eligibility and written informed consent, patients will be included into the study. Patients will be randomised into the control or intervention group. Patient characteristics and clinical variables will be collected at baseline, and 2 weeks and 9 months after discharge. Echocardiography, ECG, and laboratory analysis will be performed at baseline during hospitalisation and at the end of the study. Quality of life as part of the primary endpoint, measured with the Minnesota Living with HF Questionnaire, will be collected at baseline and at 9 months. Data about utilisation of resources will be collected prospectively and comprise components of direct costs, i.e. scheduled and non-scheduled outpatient visits and hospital admissions.

Questionnaires about self-care behaviour, anxiety, depression, compliance and medical technology assessment (MTA) will be completed before discharge, and at 4.5 and 9 months (Table 3). Patients should be included preferably as soon as possible but at least within a period of time of 14 days after discharge or after the first visit at the HF clinic with worsening HF.

**Table 3:** Questionnaires and medical assessment used in the IN TOUCH

*Questionnaires *	Baseline	2 weeks	4,5 month	9 month
Minnesota Living with Heart failure Questionnaire (MLHFQ)	•			•
Revised Heart failure Compliance	•			•
Disability Rating Index (VAS)	•			•
Hospital Anxiety and Depression scale (HADS)	•			•
Heart failure self-care behaviour scale (EHFScB)	•			•
Medical technology assessment (MTA)			•	•
EQ-5D	•		•	•
Satisfaction questionnaire for patients and providers				•
***Medical assessment***				
NYHA score	•	•		•
Echocardiography	•			
ECG	•	•		•
Physical examination	•	•		•
Laboratory tests	•	•		•

### Control group

#### ICT guided disease management system without telemonitoring

Patients in this group receive care guided by an ICT DM system. This system supports DM in a fully automatic way, assisting the HF nurse to optimise pharmacological and non-pharmacological treatment, evaluate treatment and adjust therapy to optimal levels, according to current HF guidelines. The patient will receive tailored education and counselling on the HF regimen, symptom management and improvement of the pharmacological and non pharmacological regimen.

The system mainly works as a computer decision support system. Based on the input of data from physical examination, medical history, questionnaires and nursing assessment, the system provides an advice to healthcare providers according to the actual guidelines, including up titration of HF medication to optimal doses.

### Intervention group

#### ICT guided disease management with telemonitoring

Patients in the DM with telemonitoring group will be treated with the above described ICT guided DM system in combination with the following integrated telemonitoring devices that will be installed at the patients' home;

• weighing scale; patients will be instructed to weigh daily.

• blood pressure meter; patients will be instructed to measure their blood pressure daily during up titration of medication.

• ECG; patients have to perform an ECG twice a week during up titration of beta-blockers.

• Health monitor; an interactive monitor collects data from the weighing scale, blood pressure meter and ECG device and will respond to the patients' collected data. Data will be directly transmitted to the DM system in the hospital.

In case of a deviation of individualised predefined ranges of weight, blood pressure or heart rhythm, the health monitor will automatically generate supplementary questions directly to the patient to evaluate the actual health situation. This data, measurements and subjective patient information (predefined multiple choice questions about HF symptoms) will be transferred to the computerised DM in the hospital. Furthermore, the health monitor will generate an advice about the non-pharmacological treatment, for example regarding compliance with fluid and sodium restriction. When the data collected by the system deviates from predefined ranges, the HF nurse will be informed automatically by mobile phone and email. In that case, the HF nurse will contact the patient by phone within two hours and further discusses symptoms and treatment. If data are outside any reference range in combination with symptoms of deterioration, patients will receive a message from the health monitor that they will be contacted by the HF nurse.

### Sample size calculation

Group sample sizes of 130 patients for the group treated with telemonitoring and ICT guided DM and 90 patients treated with ICT guided DM achieve 80% power to detect superiority for telemonitoring using a one-sided, two-sample t-test. The margin of equivalence is 0.0. The true difference between the means is assumed to be 0.8. The significance level (alpha) of the test is 0.025. The data are drawn from populations with standard deviations of - 2.0 and + 2.0. These figures are based on a publication [37] in which the effect of the combination of isosorbidedinitrate and hydralazin was investigated in patient with HF. In this study a difference of 0.4 was demonstrated regarding the composite endpoint. Using telemonitoring, we expect to find a larger difference especially in the QoL domain. It has to be addressed that no information is currently available regarding the sensitivity of the composite endpoint in a setting of new onset or worsening HF patients.

### Statistical analyses

The primary analysis will consist of a comparison of the composite endpoint scores (Table 2) between ICT guided DM in combination with telemonitoring, compared to ICT guided DM without telemonitoring. The test will be performed using a two-sample t-test, and two-sided 95% confidence intervals will be constructed to describe the treatment differences. An analysis of covariance will be used to test for the treatment effect controlling for different baseline characteristics. The results will be analysed using an intention-to-treat analysis including the full set of all randomised patients (primary efficacy population). The primary efficacy population will be analysed at endpoint for composite score. Secondary endpoints involving e.g. individual evaluation of deaths, hospitalisations, and QOL after 9 months will be analysed over the entire course of the study using appropriate methods. Two types of economic analyses will be performed: these include a cost-consequence analysis (CCA) for a disaggregated examination of resource costs and health outcomes associated with the alternative intervention; and cost-effectiveness analysis (CEA) in which the alternative intervention is examined in light of total cost per unit of health outcome. Thus CCA will be performed using the primary outcome of the study as the measure of effectiveness. For this, the annual cost per patient treated to postpone or prevent one patient experiencing a cardiovascular death or hospital admission for worsening HF within the trial will be calculated [38]. For CEA, the incremental cost-effectiveness ratios (ICERs), in terms of cost per life year gained (LYG), will be estimated given that there was a significant increase in survival with DM and telemonitoring. CEA will not be performed in case no reduction will be observed in cardiovascular or all cause mortality. In case of missing data for the composite end point score, the worst-case scenario will be assumed for the primary analyses. Patients lost to follow-up will be assumed to have died (-3) and those without a quality of life measurement will be assigned the worst score (-2).

### Study organisation

To include 220 patients in this study, 10 hospitals in the Netherlands are participating in the IN TOUCH study. The first patients are recruited in December 2009; the end of the study is expected to be in September 2012.

### Support and monitoring

The study is supported and monitored by the Trial Coordination Centre (TCC), a contract research organisation for clinical trials. Both the quality of the research data and of the intervention will be structurally monitored according to the GCP guidelines and TCC standards (ISO 9000:2001).

## Discussion

In the last decades, the introduction of ICT in healthcare promised an improved quality of care while reducing work load, and resulting in a more cost effective system. This might be realised by the use of computer guided decision support systems and telemonitoring [20,31,32,39,40]. The IN TOUCH study is the first to investigate the effect of an ICT guided DM system in combination with telemonitoring in patients with HF. In recent years there has been much research in the field of HF and DM. This has resulted in the implementation of DM programmes in the ESC guidelines [1]. The development of these guidelines has enabled the creation of computer aided decision support programms [39]. In the recent guidelines the application of ICT by DM programs is recommended. Although there has been progression in the development of ICT guided tools and these tools have become practical equipment for enhanced decision making, healthcare providers still experience great barriers in using and implementing them for several, often unclear reasons [33,34].

Mortality and morbidity are important outcomes in studies in HF patients. Quality of life becomes a more important issue for patients and may be even more important for them than survival or readmissions. Therefore, a composite primary endpoint, including quality of life, mortality and morbidity was chosen for the IN TOUCH study. Another reason for this composite endpoint is that for HF a high standard of care has been established which makes it more difficult to investigate the added effect of new therapeutic options. Beside the statistical advantage of a composite endpoint, the study becomes less costly and results of promising new treatment may occur earlier with this selected design. The strength of this study is the important role of ICT guided DM, telemonitoring, the composite endpoint, including quality of life, a strong focus on cost effectiveness and the emphasis on experienced user resistance and barriers regarding ICT tools in healthcare.

## Abbreviations

ICT: Information Communication Technology; DM: Disease Management; HF: Heart Failure; ESC: European Society of Cardiology; NYHA: New York Heart Association; MLHFQ: Minnesota Living with Heart Failure Questionnaire; QALYs: Quality Adjusted Life Years; ICER: Incremental Cost Effectiveness Ratio; MTA: Medical Technology Assessment; ECG: Electrocardiography; CCA: Cost-Consequence Analysis; CEA: Cost Effectiveness Analysis; TCC: Trial Coordination Centre

## Competing interests

The authors declare that they have no competing interests.

## Authors' contributions

RD, RJ, TJ and HH designed the study. AV drafted the article and all authors contributed to the final concept. All authors have read and approved the final manuscript.

## Pre-publication history

The pre-publication history for this paper can be accessed here:

http://www.biomedcentral.com/1472-6963/11/167/prepub
